# Climate Change-Related Environmental Exposures and Perinatal and Maternal Health Outcomes in the U.S.

**DOI:** 10.3390/ijerph20031662

**Published:** 2023-01-17

**Authors:** Ryne J. Veenema, Lori A. Hoepner, Laura A. Geer

**Affiliations:** Department of Environmental and Occupational Health Sciences, SUNY Downstate Health Sciences University, Brooklyn, NY 11203, USA

**Keywords:** climate change, perinatal and maternal health, birth outcomes, mental health, temperature, air pollution, natural disasters

## Abstract

Purpose: Climate change poses one of the greatest risks to human health as air pollution increases, surface temperatures rise, and extreme weather events become more frequent. Environmental exposures related to climate change have a disproportionate effect on pregnant women through influencing food and water security, civil conflicts, extreme weather events, and the spread of disease. Our research team sought to identify the current peer-reviewed research on the effects of climate change-related environmental exposures on perinatal and maternal health in the United States. Design and Methods: A systematic literature review of publications identified through a comprehensive search of the PubMed and Web of Science databases was conducted using a modified Preferred Reporting Items for Systematic Reviews and Meta-Analyses (PRISMA) approach. The initial search across both databases identified a combined total of 768 publications. We removed 126 duplicates and 1 quadruplet, and the remaining 639 publications were subjected to our pre-set inclusion and exclusion criteria. We excluded studies outside of the United States. A total of 39 studies met our inclusion criteria and were retained for thematic analysis. Findings: A total of 19 studies investigated the effect of either hot or cold temperature exposure on perinatal and maternal health outcomes. The effect of air pollution on perinatal outcomes was examined in five studies. A total of 19 studies evaluated the association between natural disasters (hurricanes, flash floods, and tropical cyclones) and perinatal and maternal health outcomes. High and low temperature extremes were found to negatively influence neonate and maternal health. Significant associations were found between air pollutant exposure and adverse pregnancy outcomes. Adverse pregnancy outcomes were linked to hurricanes, tropical cyclones, and flash floods. Conclusions: This systematic review suggests that climate change-related environmental exposures, including extreme temperatures, air pollution, and natural disasters, are significantly associated with adverse perinatal and maternal health outcomes across the United States.

## 1. Introduction

It is unequivocal that climate change poses one of the greatest risks to human health in the 21st century and beyond. Agricultural processes, land usage, the consumption of fossil fuels, such as coal and oil, and other human activities are responsible for the drastic increase in greenhouse gases in the atmosphere. Greenhouse gases, including methane, nitrous oxide, carbon dioxide, and fluorinated gases, trap energy radiating from Earth’s surface in the atmosphere. This energy is returned to the earth, where it is absorbed, resulting in increased surface temperatures. The Intergovernmental Panel on Climate Change (IPCC), the United Nations group dedicated to conducting regular assessments on climate change, states that it is extremely unlikely that the changes in the global climate could have resulted without human activities. Using measurements of past and ongoing global greenhouse gas emissions, the IPCC states with high confidence that anthropogenic warming is currently increasing at 0.2 degrees Celsius per decade [1]. In the first part of the Sixth Assessment Report, Climate Change 2021: The Physical Science Basis, the IPCC indicates that global temperatures will exceed 1.5 and 2.5 degrees Celsius above pre-industrial levels during the 21st century unless greenhouse gas emissions are drastically reduced [2].

Expected changes in the climate system include an increase in the frequency and intensity of hot temperatures, heavy precipitation, and droughts [2]. In a disastrous feedback loop, air pollution is both a driver of climate change and exacerbated by it. The higher temperatures of climate change increase the use of electricity to power fans and air conditioners to keep cool, leading to an increased release of greenhouse gases and further atmospheric warming. Additionally, as the Earth warms, there is an increased demand for moisture by the atmosphere from land and water bodies. The subsequent evaporation leads to dry soils and vegetation. Reduced air circulation and drought conditions increase the likelihood of wildfires, further contributing to the increased harmful particulate matter in the air [3]. The longer warm seasons associated with climate change also causes an increase in allergenic air pollutants, such as mold and pollen. As a result of climate change, we can expect more extreme weather events including heat waves, tropical cyclones, hurricanes, tropical storms, and flash flooding.

In the United States, the increased frequency and duration of extreme temperatures, drought leading to wildfires, and high precipitation events, are projected into the future [4]. Pregnant women and developing fetuses are uniquely vulnerable to the health impacts of these and other climate change-related events. The American College of Obstetricians and Gynecologists (ACOG), the professional organization dedicated towards the advancement of women’s healthcare, acknowledges that environmental exposures related to climate change have a disproportionate effect on women’s health through influencing food and water security, civil conflicts, extreme weather events, and the spread of disease. The ACOG states that all these factors place women at risk for disease, malnutrition, sexual violence, poor mental health, lack of reproductive control, negative obstetric outcomes, and death [5].

Our research team sought to identify the current peer-reviewed research concentrated on understanding how climate change is affecting perinatal and maternal health by focusing on three major climate change-related environmental exposure subgroups: extreme heat, air pollution, and natural disasters in the United States.

## 2. Design and Methods

### 2.1. Search Strategy

In an effort to obtain a comprehensive review of the published literature, we utilized a modified Preferred Reporting Items for Systematic Reviews and Meta-Analyses (PRISMA) approach for conducting the systematic review of the literature [6]. The PRISMA method was created in 1999 to improve the quality of systematic reviews and meta-analyses through establishing clear guidelines for identifying and screening studies as well as for reporting and interpreting results.

As recommended by the PRISMA statement, our research team collaborated to design a protocol that would capture the available peer-reviewed studies on the impact of climate related natural disasters on perinatal and maternal health outcomes. To adequately identify the current published literature, we used Web of Science and PubMed as the two search databases. The topic of climate change was searched for using the terms “climate change”, “global warming” and “climatic processes”. The three subgroups of environmental exposures included in the search were “extreme temperature”, “air pollution”, and “natural disaster”. Extreme temperature as a subgroup was searched for using the terms “temperature”, “hot temperature”, “heat exposure”, “heat event”, and “heat wave”. Air pollution as a subgroup was searched for using the terms “wildfire smoke”, “air pollution”, “ozone”, and “particulate matter”. Natural disaster as a subgroup was searched for using the terms “disasters”, “natural disasters”, and “extreme weather events”. Perinatal and maternal health were included in the search using the terms “pregnancy”, “pregnancy outcome”, “pregnancy complications”, “perinatal mortality”, “birth weight”, “birth outcome”, “fetal health”, and “gestational age”. Refer to Table 1.

The initial search across both databases identified a combined total of 768 publications. We removed 126 duplicates and 1 quadruplet, and the remaining 639 publications were subjected to our pre-set inclusion and exclusion criteria. A total of 39 studies met our inclusion criteria and were retained for thematic analysis.

### 2.2. Inclusion and Exclusion Criteria

**Figure 1:**
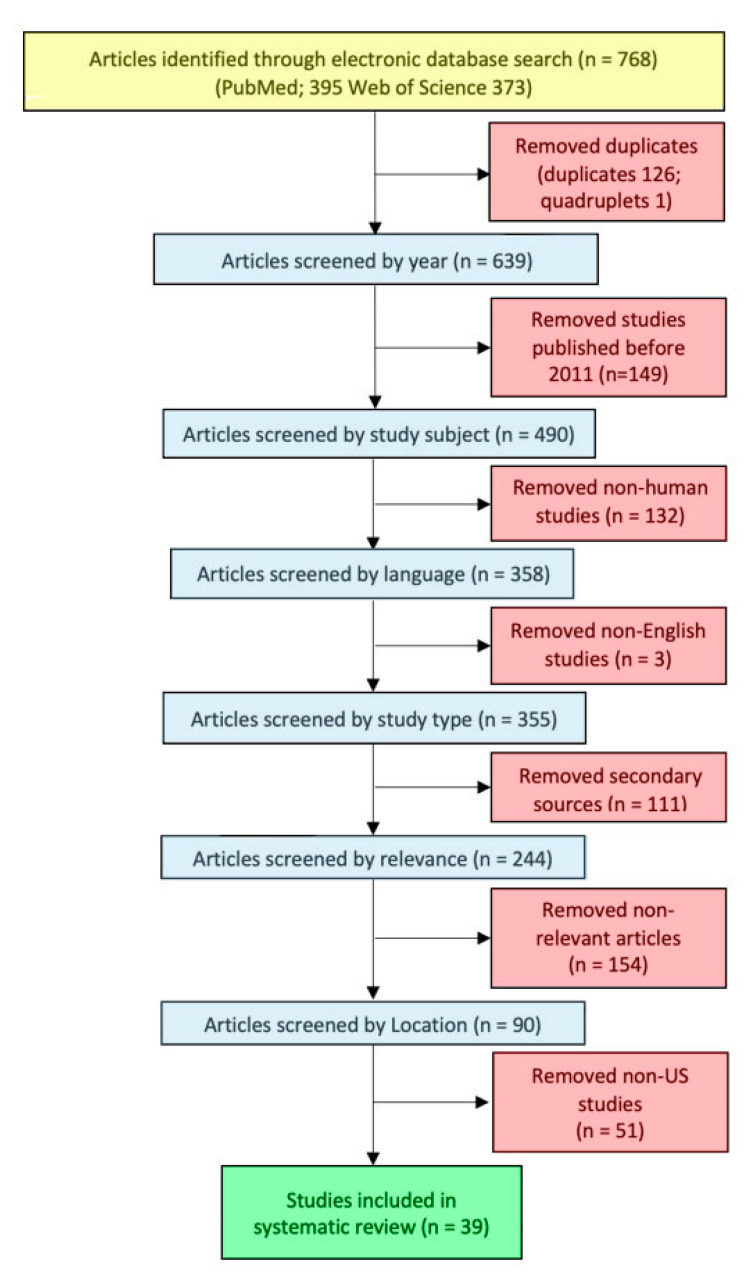
Flowchart of study selection for the systematic review.

Prior to initiating the literature search, our research team clearly defined our inclusion and exclusion criteria. To ensure relevance to present-day threats of climate change, studies published within the past 10 years were included in the study (2011–2021). Our research team limited inclusion to only research studies focused on human health outcomes. All non-human studies, including laboratory-based animal studies, were excluded from the analysis. We included primary sources published in peer-reviewed journals including cross-sectional and longitudinal studies (retrospective and prospective). Expert committee commentaries and opinion papers were not included in the study. Secondary sources, including narrative reviews, systematic reviews, and meta-analyses, were excluded. Only studies published in English were included. To be included in the review, the publication must have focused on the impact of climate change-related environmental exposures on perinatal or maternal health. Titles and abstracts were screened, and relevant studies were identified. Articles were limited to studies based in the United States. Figure 1 illustrates the implementation of our pre-set inclusion and exclusion criteria for selecting studies for the systematic review.

### 2.3. Data Extraction and Analysis

For each publication, key study and participant characteristics were collected including the study location, population, aim, and findings. We synthesized the data in a narrative format and utilized thematic analysis to identify patterns across the studies.

## 3. Results

### 3.1. Study Characteristics and Findings

A total of 19 studies from the United States investigated the effect of either hot or cold temperature exposure on birth and maternal health outcomes (Table 2). The effect of air pollution, as it relates to climate change, on birth outcomes and maternal health was examined in five studies in the United States (Table 3). A total of 19 studies evaluated the association between natural disasters and birth and maternal health outcomes in the United States (Table 4).

#### 3.1.1. Extreme Temperature Exposure Studies

The studies examined maternal exposure to both extreme cold and hot temperatures. Perinatal and maternal health outcome measures included early term birth, preterm birth, stillbirth, preterm premature rupture of membranes, low birth weight, small for gestational age, birth weight, cardiovascular events at labor and delivery, gestational age, and health worker awareness. Measures of gestational length (gestational age, early term birth, preterm birth) were assessed in six studies [7,15,18,20,22,24]. A significant association between heat exposure and either reduced gestational age, increased risk of early term birth, or increased risk of preterm birth was found in four of six studies [7,15,20,22]. Measures of birth weight (small for gestational age (SGA), birth weight, and low birth weight (LBW)) were assessed in five studies [9,11,16,18,21]. All five studies identified a significant relationship between heat exposure and either reduced birth weight, small for gestational age, or low term birth weight [9,11,16,18,21]. Cold and hot temperature exposures were associated with reduced birth weight, small for gestational age, or low term birth weight in four studies [11,16,18,21]. Lin and Zhang found that exposure to either extreme cold or extreme hot temperature was negatively associated with birth weight [16]. Ha et al. illustrated that extreme cold or extreme hot temperature exposure was associated with increased low term birth weight risk [11]. Ngo et al. found that exposure to an extra day with an average temperature either less than 25 degrees Fahrenheit or greater than 85 degrees Fahrenheit was associated with reduced birth weight [18]. Sun et al. found that low and high ambient temperature exposures were associated with low term birth weight [21]. High ambient temperature exposure was also associated with risk of SGA [21]. All three studies that examined the effect of extreme temperature exposure on risk of stillbirth found a significant association [8,12,19]. Ha et al. showed that exposure to either extreme cold or extreme hot temperatures was associated with an increased risk of still birth [12]. Birth defect outcome measures were assessed in two studies, with both studies identifying a significant association between either cold or hot temperature exposure and one or more congenital birth defects [23,25]. The Ha et al. study showed that exposure to elevated temperatures was associated with an increased risk of preterm premature rupture of membranes (PROM) [14]. Cil and Cameron showed that heat wave exposure was associated with an increased risk of abnormal conditions in the newborn and adverse maternal health conditions [10]. Monteblanco and Vanos (2021) found that maternal health workers were generally unaware of their patients’ vulnerability to adverse health outcomes from extreme heat exposures [17]. Ha et al. found that elevated temperatures were associated with an increased risk of a mother having a cardiovascular event, such as cardiac arrest or stoke, at the time of labor and delivery [13].

#### 3.1.2. Air Pollution Exposure Studies

The primary aim of three out of the five studies was to examine the effect of air pollutant exposures on perinatal and maternal health outcomes with one study examining the joint effects of heat waves and air pollutant exposures [11,22,26]. Two studies considered air pollutants as potential confounders or effect modifiers when examining the relationship between hot or cold temperature exposure and birth outcomes [9,19]. Air pollutants examined included particulate matter with a diameter less than 2.5 or 10 microns (PM2.5 and PM10, respectively), ozone, carbon dioxide, carbon monoxide, nitrogen oxides including nitrogen dioxide, sulfur dioxide, and individual constituents of PM2.5 including elemental carbon, organic compounds, ammonium ions, sulfate particles, nitrate particles, and dust particles. The outcome measures assessed included stillbirth, measures of SGA, term LBW, and preterm birth. Two studies examined the effect of air pollutant exposures on the risk of stillbirth [19,26]. Sarovar et al. found significant associations between stillbirth and short-term exposures to sulfur dioxide, ozone, and PM10 [26]. Rammah et al. determined that the significant association between stillbirth and exposure to a 10-degree Fahrenheit increase in apparent temperature was not attenuated when adjusting for PM2.5, nitrogen dioxide, or ozone exposure [19]. Measures of birth weight (term LBW, SGA) were assessed in two studies [9,11]. Ha et al. (2017) found that elevated exposures to elemental carbon and sulfate particles were associated with an increased risk for SGA [11]. It was also found that exposures to higher concentrations of dust particles and PM10 were positively associated with term LBW, although the significance was removed after adjustment for multiple comparisons [11]. Using logistic regression, Basu et al. (2018) found elevated apparent temperature was associated with term LBW, a relationship modified by ozone exposure [9]. Sun et al. found synergistic effects from exposure to heat waves and air pollutants (PM2.5, PM10, and ozone) on the risk of preterm birth [22].

#### 3.1.3. Natural Disaster Exposure Studies

The types of natural disasters studied included hurricanes (15 studies), flash floods (3 studies), and tropical cyclones (1 study) (Table 3). Perinatal and maternal health outcome measures assessed included birth weight, LBW, live birth rates, length of gestation, preterm birth, male to female birth ratio, maternal mental health, availability and usage of maternity care and support services, hypertensive disorders of pregnancy, gestational diabetes mellitus, induction of labor, emergency department visits for pregnancy complications, and composite measures of maternal and neonatal morbidity. Eight studies examined the relationship between a natural disaster exposure and a measure of gestational length [27,30,32,35,36,37,41,43]. Four of six studies found a significant relationship between hurricane exposure and either reduced gestational age or preterm birth [27,32,35,36]. Sun et al. identified a significant relationship between tropical cyclone exposure and preterm birth [43]. Hilmert et al. determined that the length of gestation was not associated with distance from or timing of a flash flood [37]. Six studies examined the relationship between a natural disaster exposure and a measure of birth weight (SGA, birth weight, LBW) [30,33,35,36,37,41]. Three of five studies found a significant relationship between hurricane exposure and either reduced birth weight, SGA, or LBW [35,36,41]. In hierarchical linear regression, Hilmert et al. showed that birth weight decreased as distance from a flash flood decreased for pregnancies exposed earlier in gestation [37]. Five studies, three hurricane and two flash flood exposure studies, examined the relationship between disaster exposure and maternal mental health outcomes [28,29,31,39,40]. Oni et al. identified positive relationships between Hurricane Katrina exposure experience and PTSD and depression [39]. Using questionnaires, Giarratano et al. found that life for pregnant women in New Orleans was still very disrupted 5 to 7 years post Hurricane Katrina [31]. Brock et al. identified that flash flood exposure was positively correlated with maternal depressive symptoms and well-being [29]. Increased support was shown to weaken the association between flash flood stress and maternal depression [28]. Oni et al. found that Hurricane Katrina exposure was associated with increased perceived stress and induction of labor. Increased perceived stress predisposed mothers to pregnancy induced hypertension and gestational diabetes mellitus [39]. Two studies examined the relationship between hurricane exposure and emergency department visits due to pregnancy complications [44,45]. Total pregnancy related complications increased during Hurricane Sandy [44]. Hurricane Sandy related power outages were associated with an increase in emergency department visits for pregnancy complications [45]. Two studies examined the effect of hurricane exposure on the usage and access to maternal healthcare and support services [41,42]. The gestational month of the first prenatal care visit was found to be significantly later within the one-year period after Hurricane Michael in Florida [41]. Interviews conducted on pregnant women exposed to Hurricane Maria in Puerto Rico illustrated that women were concerned about losing their pregnancy, worried about not having access to healthcare, and fearful of negative birth outcomes including premature birth [42]. Grech et al. determined that the male to female birth ratio increased following Hurricane Katrina [34]. Two studies determined that hurricane exposure was associated with abnormal conditions of the newborn and complications of labor and delivery [30,38].

## 4. Discussion

This systematic review assessed the relationship between climate change-related environmental exposures and perinatal and maternal health outcomes. In general, the available peer-reviewed evidence supports that climate change-related environmental exposures, including extreme temperatures, air pollution, and natural disasters, have a negative impact on perinatal and maternal health outcomes. Both low and high temperature extremes were found to negatively influence perinatal and maternal health. Studies also found significant associations between adverse perinatal outcomes and exposure to various air pollutants. Adverse perinatal and maternal health outcomes were associated with hurricanes, tropical cyclones, and flash floods.

Our systematic review findings are consistent with other review articles. In a 2020 systematic review, Bekkar et al. found that 48 of 58 air pollutant exposure studies and 9 of 10 heat exposure studies showed a significant association between exposure and adverse birth outcomes [46]. In a 2015 meta-analysis, Poursafa et al. identified that an increase in temperature was associated with an increase in preterm birth rate [47]. The authors also found that the rate of preterm birth was highest following exposure to high temperatures during the January to February months [47]. Rylander et al. concluded in a 2013 review article that climate change influences maternal health by altering access to food, drinking water, and proper sanitation through increasing extreme weather events, heat exposure, migrations, and altering the patterns of disease [48].

Additional primary research studies not captured by our literature search strategy highlight the negative influence that climate change has on perinatal outcomes. A retrospective cohort study including 7585 singleton births in Barcelona between 2001 and 2005 found that exposure to a heat index exceeding the 90th, 95th, or 99th percentile the day before delivery was associated with a significant reduction in gestational age at delivery [49]. A 2019 retrospective cohort study including 535,895 singleton births in Colorado identified that wildfire smoke PM2.5 exposure over the full gestation and during the second trimester was associated with an increased risk of preterm birth [50]. Using records from 652,167 singleton births in Massachusetts from 2001 to 2015, Qiu et al. found that prenatal exposure to PM2.5 in late pregnancy reduced gestational age at delivery [51].

Harville et al. has proposed a framework that emphasizes the multidimensional nature by which natural disasters and their aftermath affect pregnant women and infant outcomes [36]. The short-term effects include physical trauma, environmental exposures, and unstable housing. The long-term effects include forced long-term relocation, disruption of family functioning, and negative economic effects on micro and macro scales. These features of a natural disaster result in barriers to healthcare access, negative behavioral changes, and increased mental stress and negative mental health outcomes [52]. Additionally, the pre-existing disparities in health and access to healthcare are exacerbated during the time of a disaster. For example, African American pregnant women were more likely than other populations to have had severe experiences of Hurricane Katrina [35].

## 5. Limitations

We found that many relevant studies were not captured with our search strategy because climate change-related search terms including climate change, climatic processes, and global warming were not linked to the study publication on PubMed or Web of Science. This highlights the importance of including one or more of these terms with a climate change-related publication to allow researchers to identify new and relevant primary research. Limiting our search to the PubMed and Web of Science databases may have prevented us from identifying relevant, multidisciplinary, and international studies found in other databases including Scopus, ScienceDirect, and JSTOR. By focusing on perinatal and maternal health, we excluded articles that measured newborn and child health outcomes, which limited our ability to assess additional outcomes of importance along the continuum of impacts throughout the lifespan.

## 6. Conclusions

Adverse perinatal and maternal health outcomes associated with not only temperature at both extremes but also extreme weather events, which are now more frequent due to climate change, must be anticipated and built into systems for adaptation and response at all levels of prevention and care. Climate-related perinatal and maternal health outcomes would benefit from re-framing to capture the full impact of the climate across the trajectory of maternal and child health to include extreme temperatures, air pollution and natural climate-related disasters. There is also a need to identify and apply comprehensive frameworks to encompass secondary pathways/factors that can exacerbate the immediate climate-related exposures, including the lack of access to necessary resources and interruptions in access to care for the most vulnerable. Not surprisingly, social determinants play a major role in vulnerability to climate impacts and the inequity of exposure. Immediate attention is needed to further research in this area as well as to facilitate action from policymakers on prevention, preparedness, and adaptation. These activities should entail engagement by clinical care providers and a plethora of local agencies and organizations around education concerning the risks of climate-related exposures and the provision of assistance, along with support and resources for preparedness and response.

## Figures and Tables

**Table 1:** Search Strategy.

Group	Topic	Terms	PubMed	Web of Science
A	Climate Change	climate change OR global warming OR climatic processes	108,017	491,785
B	Environmental Exposure	disasters OR natural disasters OR extreme weather events OR wildfire smoke OR air pollution OR ozone OR particulate matter OR temperature OR hot temperature OR heat exposure OR heat event OR heat wave	1,247,159	4,290,162
C	Maternal Health	pregnancy OR pregnancy outcome OR pregnancy complications OR perinatal mortality OR birth weight OR birth outcome OR fetal health OR gestational age	1,197,108	633,994
D		A + C	748	777
E		B + C	22,645	9737
F		A + B + C	395	373

**Table 2:** Temperature Exposure Studies (*n* = 19).

Author, Year, Citation Number	Location	Temperature Exposure	Perinatal and Maternal Outcome
Barreca, A. and Schaller, J., 2020 [7]	United States	Heat exposure; air conditioning effects	Gestational days
Basu, R., et al., 2016 [8]	California	Mean apparent temperature	Stillbirth
Basu, R., et al., 2018 [9]	California	Apparent temperature; air pollutants as confounders or effect modifiers	Term low birth weight
Cil, G., and Cameron, T.A., 2017 [10]	United States	Heat waves	Abnormal conditions of the newborn and adverse maternal health conditions
Ha, S., et al., 2017 [11]	United States	Temperature extremes; air pollutants	Small for gestational age; term low birth weight
Ha, S., et al., 2017 [12]	United States	Acute and chronic extreme temperature exposures	Stillbirth
Ha, S., et al., 2017 [13]	United States	Ambient temperature	Cardiovascular event during labor and delivery
Ha, S., et al., 2018 [14]	United States	Ambient temperature	Preterm; premature rupture of membranes
Huang, M.J., et al., 2021 [15]	United States	Heat waves	Preterm birth and early term birth
Lin, G. and Zhang, T.L., 2012 [16]	United States	National Climatic Data Center Summary of the Day Data weather variables	Birthweight
Monteblanco, A.D., and Vanos, J.K., 2021 [17]	El Paso, Texas	Heat exposure	Awareness of health risks of extreme heat exposure
Ngo, N.S. and Horton, R.M., 2016 [18]	New York	Extreme temperatures	Birth weight and gestational age
Rammah, A., et al., 2019 [19]	Harris County, Texas	Apparent temperature; air pollutants	Stillbirths
Smith, M.L., et al., 2020 [20]	Minnesota	Heat exposure	Preterm birth
Sun, S., et al., 2019 [21]	United States	Ambient temperature exposure by trimester	Small for gestational age; birth weight
Sun, Y., et al., 2020 [22]	California	Combined effects of heat waves, air pollution and green space exposure	Preterm birth
Van Zutphen, A.R., et al., 2014 [23]	New York	Examined the relationship between extreme winter temperatures and birth defects	Birth defects
Yu, X., et al., 2018 [24]	Puerto Rico	Weather variables included monthly precipitation intensity, number of days with ≥ 25 mm rain in the month, monthly average temperature, monthly precipitation frequency (number of days with > −32.2 degrees Celsius in the month), storm events (heavy rain, hurricane, tornado and tropical storm), and flood events (flash flood and flood)	Preterm birth
Zhang, W., et al., 2019 [25]	United States	Ambient temperature changes and maternal heat exposure	Congenital heart defects

**Table 3:** Air Pollution Exposure Studies (*n* = 5).

Author, Year, Citation Number	Location	Air Pollutants	Perinatal Outcome
Basu, R., et al., 2018 [8]	California	PM2.5, ozone, carbon monoxide, nitrogen dioxide, and sulfur dioxide	Term low birth weight
Ha, S.D., et al., 2017c [14]	United States	Carbon monoxide, nitrogen oxides, ozone, PM2.5, PM10, sulfur dioxide, and individual constituents of PM2.5 including elemental carbon, organic compound, ammonium ions, sulfate particles, nitrate particles, and dust particles	Small for gestational age and term low birth weight
Rammah, A., et al., 2019 [19]	Texas	PM2.5, nitrogen dioxide, and ozone	Stillbirth
Sarovar, V., et al., 2020 [26]	California	PM2.5, PM10, ozone, nitrogen dioxide, sulfur dioxide, and carbon dioxide	Stillbirth
Sun, Y., et al., 2020 [22]	California	PM2.5, PM10, ozone, and nitrogen dioxide	Preterm birth

**Table 4:** Natural Disaster Exposure Studies (*n* = 19).

Author, Year, Citation Number	Location	Disaster Type	Perinatal and Maternal Outcome
Antipova, A., and Curtis, A., 2015 [27]	Louisiana	Hurricane	Birthweight; preterm deliveries
Brock, R.L., et al., 2014 [28]	Iowa	Flash flood	Maternal mental health
Brock, R.L., et al., 2015 [29]	Iowa	Flash flood	Maternal mental health
Currie, J., and Rossin-Slater, M., 2013 [30]	Texas	Hurricane	Birthweight; newborn abnormal conditions; labor and delivery complications
Giarratano, G.P., et al., 2019 [31]	Louisiana	Hurricane	Maternal mental health
Grabich, S.C., et al., 2016 [32]	Florida	Hurricane	Extremely preterm and preterm delivery
Grabich, S.C., et al., 2017 [33]	Florida	Hurricane	Birthweight
Grech, V., et al., 2015 [34]	Alabama, Florida, Louisiana, Mississippi	Hurricane	Male:female birth ratio
Harville, E.W., et al., 2015 [35]	Louisiana	Hurricane	Birthweight
Harville, E.W., et al., 2020 [36]	Alabama, Louisiana, Mississippi	Hurricane	Birthweight
Hilmert, C.J., et al., 2016 [37]	Fargo, North Dakota	Flash flood	Birthweight
Mendez-Figueroa, H., et al., 2019 [38]	Texas	Hurricane	Composite maternal and neonatal morbidity
Oni, O., et al., 2012 [39]	Louisiana	Hurricane	Maternal mental health
Oni, O., et al., 2015 [40]	Louisiana	Hurricane	Maternal stress and pregnancy complications
Pan, K., et al., 2021 [41]	Florida	Hurricane	Birthweight; maternity care and support services
Silva-Suarez, G., et al., 2021 [42]	Puerto Rico	Hurricane	Maternity care and support services
Sun, S., et al., 2020 [43]	United States	Tropical cyclone	Preterm birth
Xiao, J., et al., 2019 [44]	New York	Hurricane	Emergency department visits for pregnancy complications
Xiao, J, et al., 2021 [45]	New York	Hurricane	Emergency department visits for pregnancy complications

## Data Availability

Not applicable.
